# Coarse particulate matter (PM_2.5–10_) in Los Angeles Basin air induces expression of inflammation and cancer biomarkers in rat brains

**DOI:** 10.1038/s41598-018-23885-3

**Published:** 2018-04-09

**Authors:** Julia Y. Ljubimova, Oliver Braubach, Rameshwar Patil, Antonella Chiechi, Jie Tang, Anna Galstyan, Ekaterina S. Shatalova, Michael T. Kleinman, Keith L. Black, Eggehard Holler

**Affiliations:** 1 Department of Neurosurgery, Cedars-Sinai Medical Center, Los Angeles, 90048 USA; 2 Genomics Core, Cedars-Sinai Medical Center, Los Angeles, 90048 USA; 3 Department of Community and Environmental Medicine Air Pollution Health Effects Laboratory, University of California, Irvine, 92697 USA; 4 Institut für Biophysik und Physikalische Biochemie der Universität Regensburg, Regensburg, 93040 Germany

## Abstract

Air pollution is linked to brain inflammation, which accelerates tumorigenesis and neurodegeneration. The molecular mechanisms that connect air pollution with brain pathology are largely unknown but seem to depend on the chemical composition of airborne particulate matter (PM). We sourced ambient PM from Riverside, California, and selectively exposed rats to coarse (PM_2.5–10_: 2.5–10 µm), fine (PM_<2.5_: <2.5 µm), or ultrafine particles (UFPM: <0.15 µm). We characterized each PM type via atomic emission spectroscopy and detected nickel, cobalt and zinc within them. We then exposed rats separately to each PM type for short (2 weeks), intermediate (1–3 months) and long durations (1 year). All three metals accumulated in rat brains during intermediate-length PM exposures. Via RNAseq analysis we then determined that intermediate-length PM_2.5–10_ exposures triggered the expression of the early growth response gene 2 (EGR2), genes encoding inflammatory cytokine pathways (IL13-Rα1 and IL-16) and the oncogene RAC1. Gene upregulation occurred *only* in brains of rats exposed to PM_2.5–10_ and correlated with cerebral nickel accumulation. We hypothesize that the expression of inflammation and oncogenesis-related genes is triggered by the combinatorial exposure to certain metals and toxins in Los Angeles Basin PM_2.5–10_.

## Introduction

Air pollution leads to well-documented cardiovascular and respiratory problems^1,2^, brain cancer, and neurological disorders including strokes, Alzheimer’s, and Parkinson’s disease^3–7^. To better understand the biological mechanisms that underlie pollution-associated brain disorders, we must identify disease causing components in air pollution mixtures; these include gases, particulate matter (PM), trace metals, and organic compounds. We focus on the synergistic interplay between PM, trace metals and toxins in Los Angeles Basin ambient air pollution.

In humans, exposures to high levels of air pollution are associated with chronic neuroinflammation and neurodegeneration^8,9^. Brain tissue samples from individuals residing in Mexico City contained increased numbers of infiltrating monocytes, activated microglia, increased expression of interleukin 1 beta, blood-brain barrier (BBB) damage, endothelial cell activation and prefrontal cortical lesions^9,10^. Moreover, increased levels of inflammatory biomarkers, deposits of alpha-synuclein, amyloid beta protein and hyper-phosphorylated tau protein, collectively indicative of Alzheimer’s disease, have been documented in brains from urban residents^9,11–13^.

Animals show similar responses to air pollution. Mexico City stray dogs had elevated concentrations of vanadium and nickel in cortical and olfactory bulb neurons. Amyloid beta protein depositions were also detected^14^. Experimentally, it has been shown that prolonged exposures to diesel exhaust cause neuroinflammation in rats^15^, while nanoscale PM from Los Angeles urban traffic induced oxidative stress and inflammation in the olfactory epithelium and the brain^16^. Exposure to vehicle exhaust also increases anxiety and depression-like behaviors in rats^17^, and accelerates plaque formation in a mouse model of Alzheimer’s disease^18^.

In the present study, airborne PM were defined as either coarse (PM_2.5–10_: 2.5–10 μm), fine (PM_<2.5_: <2.5 μm) or ultrafine particulate matter (UFPM: <0.25 μm). PM_<2.5_ also contained UFPM. Each type of PM has a distinct composition and mediates different effects on organ health. UFPM deposit deep in the lungs and are small enough to cross epithelial barriers into the circulation. From there, UFPM can impact distal organs, including the heart and brain^3^, and enter intracellular compartments to disrupt normal cell function^19,20^. Coarse PM, on the other hand, deposit via inertial impactation in the upper respiratory system and may be partially removed via mucociliary clearance or ingestion/sequestration in lymphoid and intestinal tissues. Coarse particles probably do not enter the circulatory system directly, but trace metals, endotoxins and other soluble compounds present on coarse PM can leach into the fluid lining of the airways. Leached metals trigger the production of reactive oxygen species via Fenton-like reactions^21^ and soluble toxins can activate molecular signaling cascades that trigger tissue inflammation^22^. Pro-inflammatory cytokines, monocytes and macrophages are subsequently released into the systemic circulation^3^, and if sustained, can trigger BBB damage^23,24^, nervous system inflammation^25^ and the expression of cancer-related genes in brain tissue^26^. Many studies that have investigated PM effects on organ health defined PM_10_, as per regulation, as any particulate matter sized ≤10 μm. PM_10_ therefore also contains PM_<2.5_ and UFPM. To better understand how individual PM types affect our health, it is instructive to separate the coarse and fine particles. We used a material impactor to separate coarse from fine PM and then fractionated the fine PM into PM_<2.5_ and UFPM aerosols. Therefore, PM_2.5–10_ in our study contains only 2.5–10 μm sized particles.

Below we describe our observations on the association between toxic metals found in different PM types with the expression of inflammation and oncogenes in rat brains. Atomic emission spectroscopy was first used to analyze the metal contents of PM_2.5–10_, PM_<2.5_ and UFPM in ambient air from the Los Angeles Basin. We then exposed rats for short (2 weeks), intermediate (1–3 months) and long (1 year) time periods separately to each PM type. We demonstrate that intermediate exposures to PM_2.5–10_ and UFPM lead to cerebral metal accumulation. However, upregulation of inflammation and tumorigenesis biomarkers was only observed following exposures to PM_2.5–10_, not UFPM. We explain this finding by comparing the unique toxicological profiles of PM_2.5–10_ and UFPM; both contain metals, but only PM_2.5–10_ specifically correlates with ambient endotoxin concentrations in Southern California^27,28^. This suggests that combinatorial exposures to multiple toxins are necessary to trigger certain molecular events that precede inflammation and tumorigenesis.

## Results

We exposed rats separately to either PM_2.5–10_, PM_<2.5_, UFPM or filtered air. Via inductively coupled plasma atomic emission spectroscopy (ICP-AES), we first analyzed the metal content of our PM samples and then asked if these metals accumulated in the brains of rats that were exposed to PM.

Six metals, namely cadmium, cobalt, lead, nickel, vanadium, and zinc, were detected via ICP-AES in our PM samples. Only nickel (Ni), cobalt (Co), lead, and zinc (Zn) were identifiable in all types of PM (Table 1). The Ni content ranged from 0.0025 to 0.012 μg/m^3^, the Co content from 0.0016 to 0.0037 μg/m^3^, the lead content from 0.0023 to 0.034 μg/m^3^, and the Zn content from 0.042 to 0.265 μg/m^3^ (Table 1). Zn was thus the most prevalent metal in our PM samples.

**Table 1:** Metal content in PM polluted air in μg/m^3^.

Element	PM_2.5–10_ (μg/m^3^/PM)	PM_<2.5_ (μg/m^3^/PM)	UFPM (μg/m^3^/PM)
Antimony	n.d.	n.d.	n.d.
Arsenic	n.d.	n.d.	n.d.
**Cadmium**	n.d.	**0.0022**	**0.0011**
**Cobalt**	**0.0026**	**0.0037**	**0.0016**
**Lead**	**0.0023**	**0.0343**	**0.011**
**Nickel**	**0.0025**	**0.012**	**0.0053**
Palladium	n.d.	n.d.	n.d.
Platinum	n.d.	n.d.	n.d.
Rhodium	n.d.	n.d.	n.d.
**Vanadium**	n.d.	**0.0016**	**0.0025**
**Zinc**	**0.042**	**0.2650**	**0.078**

Heavy metal concentrations in PM sourced from Riverside, California. Metals present at or above the detection level are in shown in bold. The abbreviation n.d. indicates no detection.

**Figure 1:**
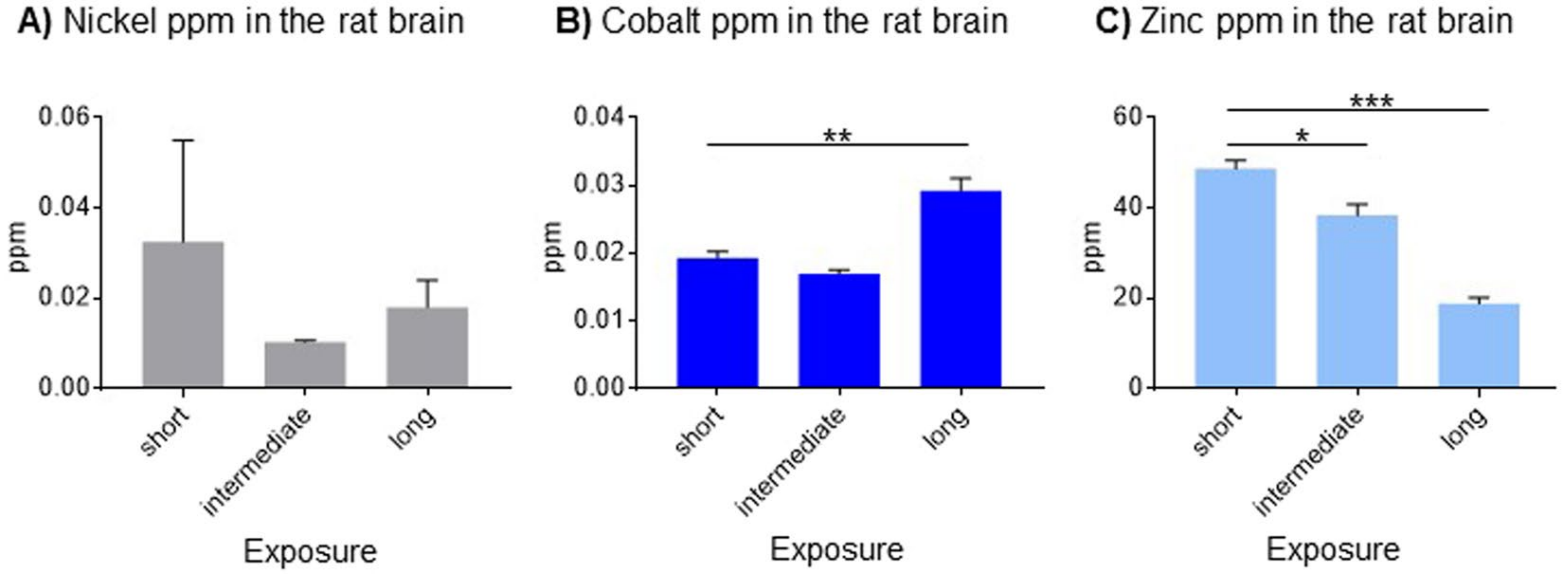
Metal content in rat brains after exposures to filtered air. Levels of (**A**) nickel were approximately stable, while (**B**) cobalt accumulated, and (**C**) zinc dissipated. Data are shown as mean + S.E.M. Statistical significance is indicated as p < 0.05 (*), p < 0.001 (**) and p < 0.0001 (***).

Rats in all experimental groups, including those exposed to filtered air, had detectable levels of Ni, Co and Zn in their brains (Fig. 1). The Ni content remained approximately constant over 12 months (Fig. 1A), but the Co content increased significantly after 12 months of exposures to filtered air (Fig. 1B; ANOVA: F(2,9) = 32.35, p < 0.001). The Zn content was approximately 1000-fold higher than that of Co and Ni, but showed a significant decrease after 12 months of exposures to filtered air (Fig. 1C; ANOVA: F(2, 7) = 68.52, p < 0.001). These results demonstrate that a certain amount of heavy metal accumulation (Co) and clearance (Zn) occurs in the absence of targeted PM exposures. We next compare metal contents in the brains of rats exposed to concentrated ambient PM vs. filtered air.

### Significant accumulation of Ni, Co and Zn after 1 month of PM exposures

**Figure 2:**
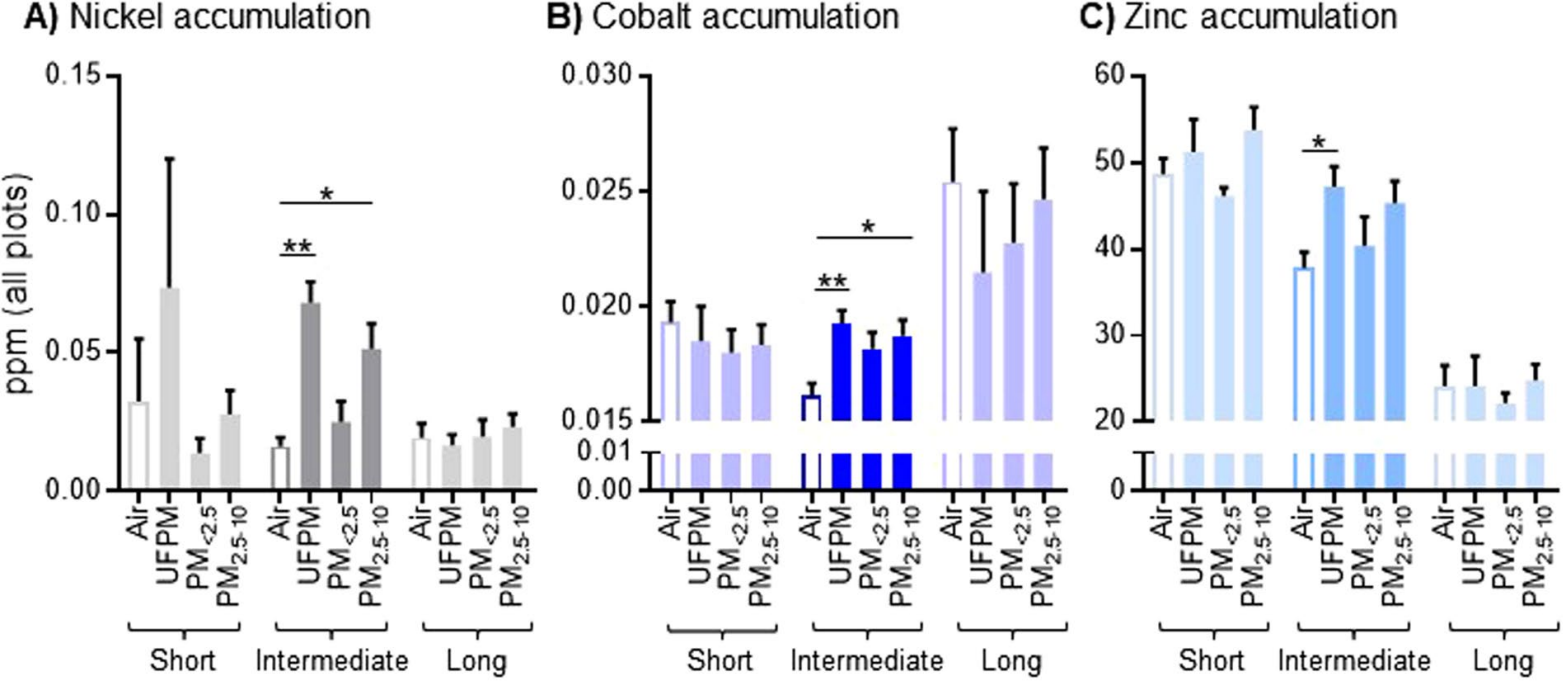
Metal accumulation in the rat brain after short, intermediate and long exposures to PM polluted air. (**A**) Ni, (**B**) Co and (**C**) Zn show significant accumulations after PM exposures of intermediate durations (1–3 months). Data are shown as mean + S.E.M. Statistical significance is indicated as p < 0.05 (*) and p < 0.001 (**).

Exposing rats to PM_2.5–10_, PM_<2.5_ or UFPM for 2 weeks did not cause significant accumulations of metals in their brains (see Fig. 2A–C short exposures). However, intermediate length exposures of 1–3 months, led to significant increases of cerebral metal contents in comparison to specimens from filtered air control groups (ANOVA_Ni_: F(3, 23) = 10.04, p = 0.0002; ANOVA_Co_: F(3, 31) = 5.93, p = 0.002; ANOVA_Zn_: F(3, 28) = 2.75, p = 0.06). Cerebral Ni levels were increased after exposures to UFPM (p < 0.001) and PM_2.5–10_ (p = 0.011, both Dunnett t-test; see Fig. 2A). Cerebral Co accumulation was similarly increased after exposures to UFPM (p < 0.001) and PM_2.5–10_ (p = 0.006, both Dunnett t-test; see Fig. 2B). Zn showed only a modest increase after exposures to UFPM (p = 0.043, Dunnett t-test; see Fig. 2C).

Interestingly, no accumulation of Ni, Co or Zn was observed after long term PM exposures of up to 12 months (Fig. 2A–C). Only a slight increase in the cerebral Ni content was observed for the PM_2.5–10_ and PM_<2.5_ exposure groups, however, this was not significant (Fig. 2A). Thus, cerebral contents of Ni, Co and Zn are significantly increased after intermediate exposures to PM_2.5–10_ and UFPM. This accumulation is transient.

### Coarse PM causes upregulation of inflammation and cancer-related genes

**Figure 3:**
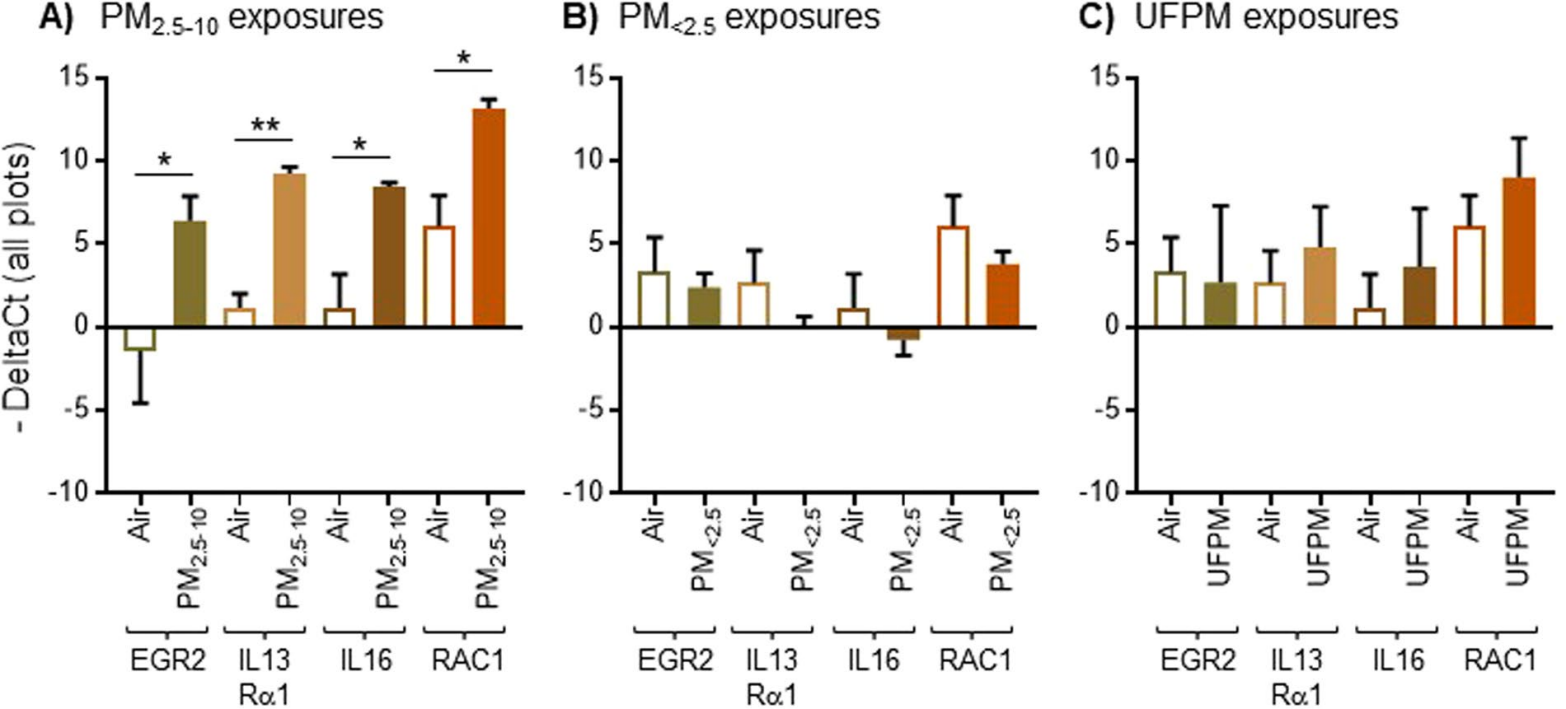
Expression of genes related to inflammation (EGR2, IL13Rα1, IL16) and cancer (RAC1) in the rat brain after intermediate exposures to PM (1 month). All genes are upregulated after intermediate-length exposures to PM_2.5–10_ (**A**) but not PM_<2.5_ (**B**) nor UFPM (**C**). Data are shown as mean + S.E.M. Statistical significance is indicated as p < 0.05 (*), p < 0.001 (**).

We next analyzed the expression levels of eight genes that are linked to neuro-endocrine responses, proliferation, inflammation, and tumor development (Table 2). None of the genes showed different expression levels after short term exposures to PM. However, after intermediate exposures to PM_2.5–10_, we observed a significant upregulation of four genes in the brains of the exposed rats (Fig. 3A). Specifically, expression of the early growth response gene-2 was significantly increased after intermediate exposures to PM_2.5–10_ (Fig. 3A; EGR2; p = 0.0012, Welch’s test and p = 0.0232 by FDR test). EGR2 is a transcription factor that modulates inflammatory responses and is known to promote the development of non-small cell lung carcinoma^29,30^. The genes encoding the interleukin (IL) receptor-13α1 (IL-13Rα1, p = 0.0009, Welch’s test and p = 0.0219 by FDR test) and the cytokine IL-16 (p = 0.0027 by Welch’s test and p = 0.0426 by FDR test) were concomitantly upregulated after intermediate exposures to PM_2.5–10_ (Fig. 3A); expression of these genes is a good indicator for inflammation and tumorigenesis^31–33^. Finally, we observed upregulation of the Ras-related botulinum toxin substrate 1 (RAC1) gene after intermediate exposures to PM_2.5–10_ (Fig. 3A; p = 0.005 by Welch’s test and p = 0.0669 by FDR test). RAC-1 activity has been associated with various types of cancer^34,35^.

**Table 2:** Primers used for Fluidigm BioMark^TM^ microfluidic qPCR gene expression analysis of rat brain tissue after exposure to PM.

Primer#	Gene (Full Name)	Gene symbol	Gene Type	Assay ID	Exon Boundary/Assay Location	Amplicon Length
1	Glycoprotein hormone, α polypeptide	Cga	Pituitary hormone	Rn01440184_m1	3-4/376	56
2	Growth hormone 1	Gh1	Peptide hormone	Rn01495894_g1	1-2/69	60
3	Prolactin	Pro	Peptide hormone, development	Rn00561791_m1	3-4/493	86
4	Thyroid Hormone, β subunit	Tshβ1	Peptide hormone	Rn00565424_m1	1-2/72	74
5	Interleukin 13 Receptor α1	IL13α1	Cytokine receptor	Rn01457340_g1	1-1/272	123
6	Interleukin 16	IL-16	Cytokine	Rn01477715_m1	18-19/3974	64
7	Ras-related botulinum toxin substrate 1	RAC-1	G-protein	Rn01412766_m1	4-5/289	86
8	Early growth response gene 2	EGR2	Transcription factor	Rn00586224_m1	1-2/512	67
9	GAPDH	GAPDH	Dehydrogenase	Rn01775763_g1	1-1/1154	174
10	Actin	Actin	Cytoskeletal protein	Rn00667869_m1	4-5/884	91

Crucially, the four above-mentioned genes were only upregulated after intermediate exposures to PM_2.5–10_. No difference in gene expression was observed in rats that received intermediate-length exposures to PM_<2.5_ (Fig. 3B) or UFPM (Fig. 3C). In addition, none of the genes were overexpressed following prolonged exposures to PM for up to one year. Instead, several genes appeared to be downregulated in PM_<2.5_ and UFPM exposure groups (Supplementary Fig. S1). Taken together, these data show that intermediate exposures to PM_2.5–10_ cause a transient expression of inflammation and cancer biomarkers in the rat brain.

### A correlation between Ni accumulation and gene upregulation

**Figure 4:**
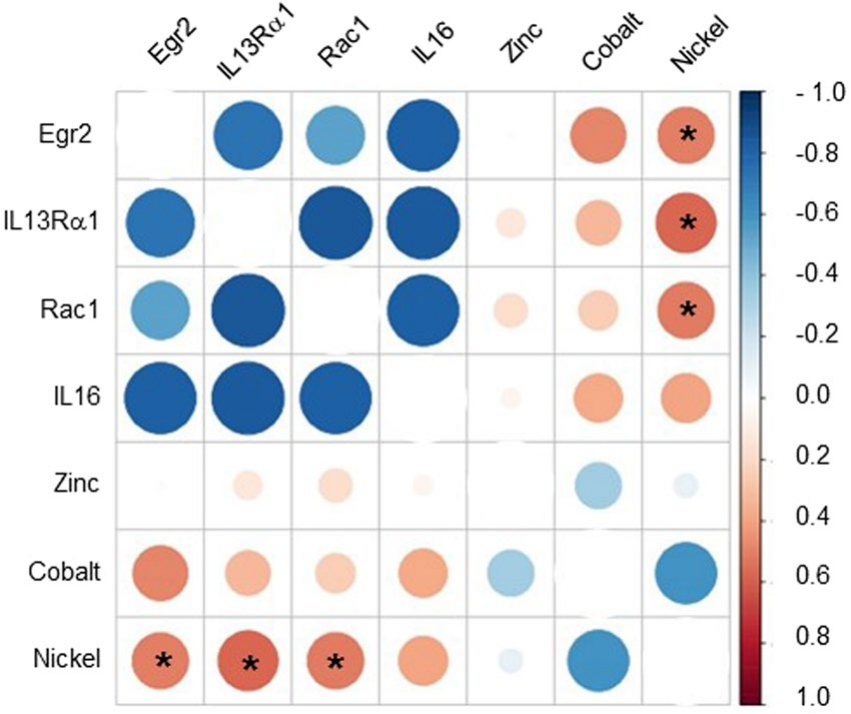
Spearman correlation between metals found in PM and genes indicative of inflammation and cancer. Significant correlations between Ni accumulation and several upregulated genes were observed and are indicated with asterisks. Co and Zn accumulation did not correlate with increased gene expression, whereby the small orange circles in the Co group may be regarded as indices for a weak correlation. The color scale indicates Spearman rho values; strong associations are colored red (rho = 1). The size of the circle also indicates the strength of the correlation, with a larger circle indicating more evidence in support of correlations (rho = 1). Statistical significance is indicated as p < 0.05 (*).

Based on our findings, we conclude that PM_2.5–10_ cause upregulation of four inflammation and cancer related genes in the rat brain. Because we also observed significant accumulations of Ni and Co following exposures to PM_2.5–10_ (i.e., Fig. 2A,B), we next asked if there was a link between these metals and gene upregulation. The resulting Spearman’s correlation analysis is shown in Fig. 4. The strength of metal accumulation to gene expression correlations are indicated by color and circle size, where a large red circle indicates a maximum correlation. Only Ni accumulation correlated consistently with upregulated gene expression. Specifically, accumulated Ni and EGR2 gene expression were significantly correlated (see asterisks in Fig. 4; rho = 0.508; p = 0.0492). Moreover, Ni and IL-13Rα1 expression were strongly correlated (rho = 0.577; p = 0.019), while RAC1 expression and Ni accumulation were also correlated (rho = 0.512; p = 0.042).

Despite indices for a correlation between accumulated Co and EGR2 expression (rho = 0.484; p = 0.067), no statistical significance was observed between Co accumulation and gene expression (but see small red circles in Co row). Finally, Zn accumulation lacked any indices for correlating with upregulated gene expression (see small or absent circles in Zn row in Fig. 4). Thus, we identify a specific association between accumulated Ni with the expression of inflammation- and cancer related genes. Ni may thus be a partial trigger of gene upregulation in response to PM_2.5–10_ exposures.

## Discussion

**Figure 5:**
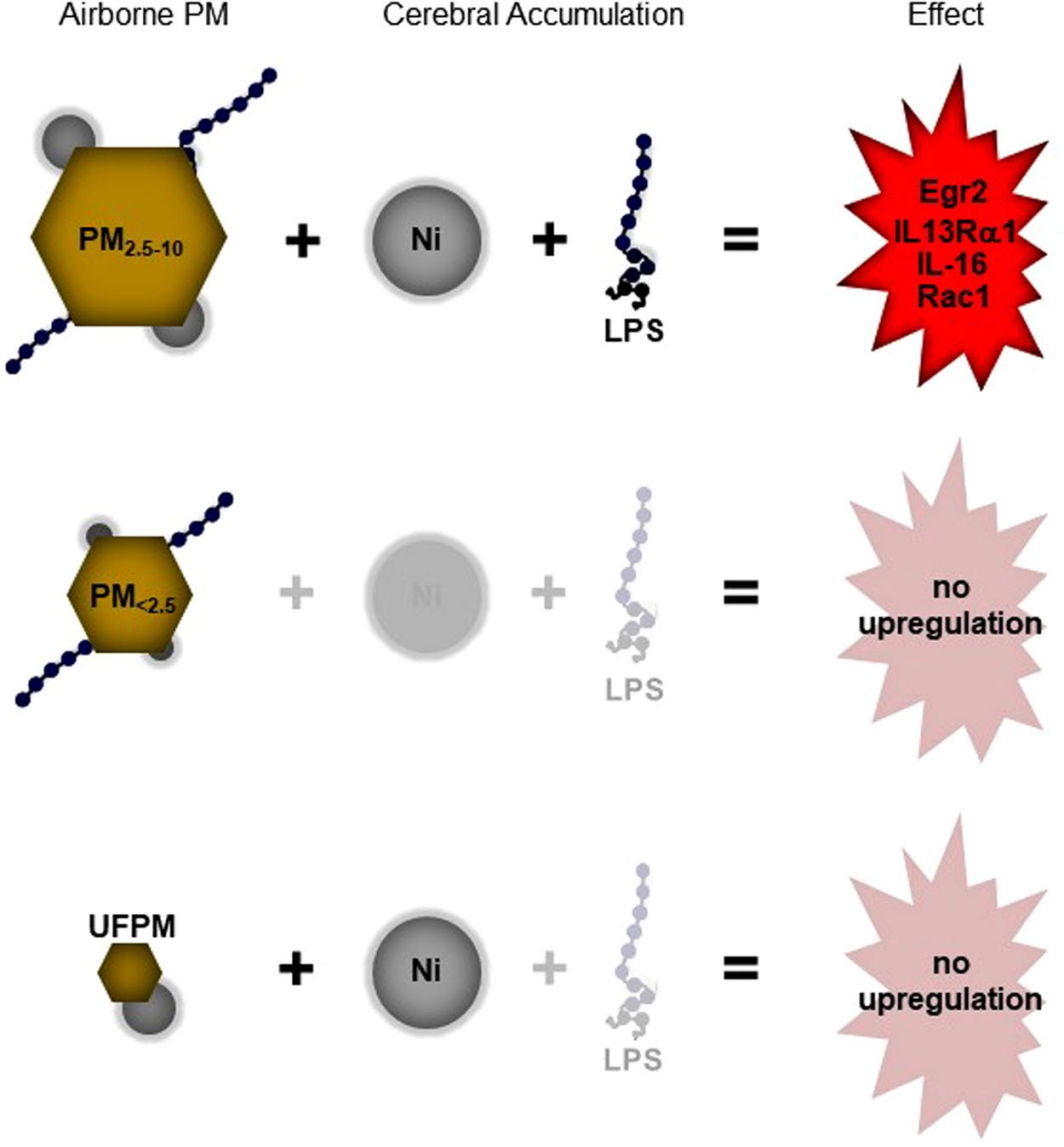
Working hypothesis to explain gene upregulation observed following PM_2.5–10_ exposures. PM_2.5–10_ contains both nickel (see Table 1) and LPS (see text) and triggers upregulation of several genes. PM_<2.5_ contain nickel (see Table 1) and LPS (see text) but presumably deposit poorly in the nasal and endotracheal airways (see text); hence no Ni accumulation was observed after PM_<2.5_ exposures. UFPM contain nickel (Table 1) but are not always associated with LPS, and do not trigger gene upregulation. Thus, the combination of Ni accumulation and LPS appears to be required to trigger gene upregulation; additional toxins and metals that are not mentioned here are likely contributing factors.

Ambient air pollution contains ubiquitous PM that vary in size and composition. Each PM type has a unique toxicological signature which may determine its interactions with molecular cascades in impact organs. We exposed rats separately to PM_2.5–10_, PM_<2.5_ and UFPM from Riverside, California. We show that PM_2.5–10_ and UFPM exposures lead to cerebral metal accumulation. However, only the PM_2.5–10_ exposures triggered upregulation of inflammation and cancer-related genes. Based on known toxicological profiles of ambient PM from Southern California, we conclude that combinatorial exposures to certain metals and toxins in PM_2.5–10_ are necessary and responsible for the expression of inflammation and cancer-related biomarkers. This hypothesis is summarized in Fig. 5 and discussed in more detail below.

### Combinatorial toxicity of metals and other toxins in PM_2.5–10_

The PM_2.5–10_ that we used in exposure experiments contained Ni (see Table 1) and led to cerebral Ni accumulation (Fig. 2A). A previous analysis of PM_2.5–10_ from Southern California concluded that endotoxin concentrations are elevated in ambient PM from Riverside^28^. Endotoxins, or bacterial lipopolysaccharides (LPS), are potent triggers of inflammatory responses in pulmonary, cardiovascular, and brain tissue^22,36–39^. We thus hypothesize that the combined exposure to metals (i.e., Ni) and LPS contained in ambient PM_2.5–10_ from Riverside triggered upregulation of EGR2, IL-16, IL-13Rα1 and RAC-1 and putatively an inflammatory response in rat brains (Fig. 5A).

No gene upregulation was observed following intermediate-length exposures to PM_<2.5_. PM_<2.5_ contain metals (see Table 1) and LPS^28^. However, PM_<2.5_ deposit poorly in rat airways. Tissue deposition of PM is governed by inertia and Brownian diffusion, which favor the deposition of micrometer-sized coarse PM and nanometer-sized UFPM, respectively. Fine PM_<2.5_ is too light for deposition by inertia and too large for absorption via Brownian diffusion, and thus accumulates only marginally in nasal^40^ and endotracheal/pulmonary airways^41,42^. Ultimately, this limits the interaction between PM_<2.5_ and biological tissues, and hence, the transfer of toxic metals and LPS to impacted organs. This would explain why we failed to observe cerebral Ni accumulation (Fig. 2) and gene upregulation (Fig. 3B) following exposures to PM_<2.5_ (Fig. 5B).

Finally, UFPM contain toxic metals (Table 1) and UFPM exposures lead to cerebral Ni accumulation (Fig. 2A). However, UFPM in Southern California may not always contain LPS^27^, and do not correlate in ambient concentration^28^ or distribution with endotoxins^43^, suggesting that endotoxins present themselves independently of UFPM. Indeed, we observed that UFPM do not trigger the expression of EGR2, IL-16, IL-13Rα1 and RAC-1 in rat brains (Fig. 5C) and we therefore conclude that (1) adequate PM deposition, (2) Ni (or other metal) accumulation and (3) LPS (or other toxin) exposures were necessary to induce the upregulation of the gene repertoire studied in this experiment.

Our findings are consistent with prior reports of synergistic toxicological effects of multiple PM components. Dong *et al*. (1996) demonstrated that upregulation of inflammatory cytokines in alveolar macrophages occurred in response to urban air and diesel exhaust, but only if air and exhaust particles contained LPS^44^. A similar result was recently published by^45^. Farina *et al*. (2013) reported greater toxicity and pathological effects of Milano-sourced PM_10_ during the summer months, when concentrations of toxic LPS are elevated^23^. More recently, Woodward *et al*.^22^ demonstrated that LPS and nano-sized PM (<0.2 μm) trigger glial inflammatory responses via activation of the toll-like 4 receptor. Twenty-five percent of the genes that were upregulated in response to either LPS or PM were shared, suggesting a synergistic interplay between PM, LPS and molecular signaling pathways^22^. Finally, Ni is one of the most frequently identified metals in airborne PM^46–48^ and multiple studies show that Ni is correlated with cardiovascular abnormalities^49,50^. However, Ying *et al*. (2013) demonstrated that Ni exposures alone are not effective in inducing cardiovascular tissue responses, and that these occur only in tissue exposed to Ni and LPS^48^. Combinatorial interactions of metals with other PM components and toxins are therefore required to induce molecular signaling pathways that induce tissue inflammation.

### How do PM_2.5–10_ affect the brain?

We showed that exposures to PM_2.5–10_ triggered the expression of genes related to inflammation and oncogenesis in rat brains. How do exposures to coarse ambient PM result in brain inflammation? Two routes may be considered. First, coarse PM deposit efficiently in superficial airways and to some degree in the lung^41,42^. Trace metals, endotoxins and other soluble compounds that are present on the coarse PM can leach into the fluids lining the airways, interact with tissue, and trigger the production of reactive oxygen species via Fenton or Fenton-like reactions (i.e., metals)^21^, or activate molecular signaling cascades that initiate tissue inflammation (i.e., LPS)^22^. Pro-inflammatory and inflammatory factors may then be released into the bloodstream to interact with- and disrupt the blood brain barrier (BBB). Additional cytokines, monocytes and macrophages can enter the brain via the disrupted BBB to trigger local inflammatory responses^3,23^. This may explain why brains from individuals in highly polluted cities have increased monocyte infiltration, activated microglia, increased interleukin activity, BBB damage and localized brain lesions^8,9,11^.

A second route for coarse PM to induce brain inflammation exists via the olfactory system. Coarse PM deposit efficiently in the rat olfactory epithelium^40^ and leach soluble metals and toxins into the nasal mucosa. Toxic metals translocate directly into olfactory sensory neurons and are transported along their axons into the olfactory bulbs^51–53^. Once inside the olfactory bulbs, metals and other UFPM may trigger local immune responses, or infiltrate upstream brain regions^16,54^ to trigger additional inflammatory responses. This presumably occurs via synergistic interactions with other toxins (e.g., LPS), cytokines and macrophages that have gained access to the brain via the same route, or via a compromised BBB, as described before. Coarse PM thus pose significant challenges to the normal function and health of the brain and it will be instructive to study the interplay between this type of pollution with molecular mechanisms of inflammation and disease in more detail.

### Inflammation and cancer-related genes as proxies for neurological disorders

Gene expression analysis of rat brains revealed changes in several genes related to inflammation following exposures to PM_2.5–10_. A previous study by our group identified changes in gene expression in rat brains after exposure to coarse PM via by RNA-seq analysis^26^. Based on these data, we investigated the expression of eight genes (Table 2) that could be affected by pollution exposures and may play important roles in the molecular changes that precede brain pathology.

We found a transient upregulation of IL16 and IL-13Rα1. Tissue expression of IL16 recruits and attracts a variety of immune response cells, including monocytes and dendritic cells^32^. The IL-13Rα1 gene, on the other hand, encodes a receptor subunit of IL13 and IL4 receptors; cytokine receptors of this variant are implicated in diverse regulatory and pathological processes, including alternative macrophage activation^55^ and neurodegenerative disorder^56^. EGR2 is an important modulator of immune responses, and acts primarily to suppress them. EGR2 has been linked to autoimmunity and various immune responses related to cancer^29,30^. Thus, the collective upregulation of IL16, IL-13Rα and EGR2 genes is a strong indication of an ongoing inflammatory process.

Activity and/or expression of the above-mentioned genes has, on multiple occasions, been linked to cancer. For example, IL-13Rα2 plays a role in the development of glioblastoma multiforme^57–60^ and is associated with human and canine astrocytoma^61^. IL-16 immunoreactivity has been correlated to increasing grades of human astrocytic brain tumors^62^ and EGR2 expression has been linked to human glioblastomas^63^. RAC1 is one of the most commonly mutated oncogenes in human cancers and has been linked to aberrant cell cycle progression and survival^64^, growth factor–induced membrane ruffling^65^ and other signaling mechanisms in glioblastoma multiforme^66^. Thus, the genes that we have found to be upregulated in response to PM_2.5–10_ are proxies that signal developing or ongoing brain inflammation and oncogenic mechanisms. Our data thus provide another putative link between PM pollution and brain pathology.

## Conclusion

In the present study we show how PM_2.5–10_ from the Los Angeles basin urban area can trigger the expression of inflammation and cancer-related biomarkers in the rat brain. Our data show that PM induced toxicity is caused by synergistic effects of metals and toxins that are uniquely present in some PM types. Our findings may be unique to the Los Angeles Basin, and may depend heavily on the air pollution composition in this region. Other large urban settlements likely contain differently composed air pollution mixtures, however, there are many examples of potentially deleterious effects of pollution exposures in major cities^13,23,67,68^. Our modern society is becoming increasingly urbanized and exposed to more and more air pollution. This underscores the need for additional research on the biology of air pollution-induced organ damage and/or a concerted political effort aimed at reducing ambient air pollution levels.

## Methods

### Particle isolation and characterization

We used the versatile aerosol enrichment system (VACES), coupled to size-selective inlets, to isolate and concentrate coarse (PM_2.5–10_: 2.5–10 µm), fine (PM_<2.5_: <2.5 µm) and ultrafine particles (UFPM: <0.25 µm) from ambient air pollution. The VACES has been described in detail in^69,70^ and a similar experimental application as for the present study in^71^. The exposure location was Riverside, CA. Riverside is downwind from Los Angeles and has high regional pollution mixed with vehicular emissions, which originate from a freeway near the exposure site. Exposures were centered around the summer months. Detailed descriptions of the exposure method are available in^26,71^.

Physical and chemical characterization of PM was conducted based on samples that were collected from filters installed upstream of the exposure chambers. PM were collected for four days collected on 25 mm Teflon filters (Zefluor 1-mm port; Pall Corporation, Ann Arbor, MI), which were weighed before and after each exposure session. Elemental and carbon contents were measured from PM collected on pre-baked quartz filters (Tissuequartz, Pall Corporation) after four cumulative days of exposures. The quartz filters were stored after each exposure session, and re-used in subsequent exposure sessions. Elemental and organic carbons were determined using the thermal MnO_2_ oxidation method by Atmoslytic Inc. (Calabasas, CA). Particle concentrations of PM_<2.5_ and UFPM were measured using a TSI 3022 Condensation Particle Counter (TSI Incorporated, Shoreview, MN). Details on PM mass and composition in this experiment are supplied in Supplementary Table S2.

### Animal exposures to PM

All animal experiments were performed in accordance with the protocols approved by the University of California at Irvine (IACUC Protocol # 2001–2242) and the Cedars-Sinai Medical Center Institutional Animal Care and Use Committee (Protocol #9121). Fisher rats aged 8–10 weeks were obtained from Harlan, Inc. (Indianapolis, IN) and fed LabDiet^®^ 5001 rodent food (PMI^®^ Nutrition International, LLC, Brentwood, MO). One hundred rats (6–10 rats per group) were used for our experiments.

Rats were exposed to PM for 5 hours daily, 4 days per week for either two weeks (short), one month (intermediate), three months (intermediate), or 12 months (long). Results from one and three month conditions were pooled for all analyses in this paper. Control experiments were carried out with a cohort of rats that were exposed to filtered air for the same durations as the rats in the PM exposure groups. Filtered air was produced by passing air through an activated carbon filter and then a HEPA filter.

### Metal content analysis

Metal analysis of PM and of brain tissue was performed by the GLP certified laboratory Exova (Santa Fe, CA) using Inductively Coupled Plasma Atomic Emission Spectroscopy (ICP-AES)^72^. PM_2.5–10_, PM_<2.5_ and UFPM were first collected at a flow rate of 0.5 L/min on 37 mm Teflon filters (PTFE 2, Gelman Science, Ann Arbor, MI). PM samples were then digested in 1 mL nitric acid (36%w/v) and 1 mL hydrochloric acid (86%w/v) for 1 hour at 110 °C on a heated plate. The samples were then allowed to cool and the digestion was resumed for another 30 minutes in 0.5 ml hydrogen peroxide (30%w/v). Samples were again cooled, internal standards were added, and then diluted to a final mass of 20 g. A total of 11 heavy metals were tested and six of them were found in detectable quantities (Table 1).

Metal analysis of brain tissue was conducted on brains that were obtained 24 hours after the last exposure. Rats were euthanized and brains were promptly harvested. Each brain was cut in half along the mid-sagittal plane to yield two equal hemispheres; one hemisphere was used for metal analysis and the other was used for molecular analysis (see below). Whole hemispheres were weighed and homogenized and treated for 1 hour at 110 °C with a mixture of 0.5 ml nitric acid (36%w/v) and 0.5 ml hydrochloric acid (86%w/v). After cooling, 0.5 ml hydrogen peroxide (30%w/v) was added, and the samples were incubated for an additional 30 minutes. Internal standards were added and the samples were then diluted with distilled water to a final mass of 5 g. Metals that were initially found in PM were then analyzed in the brain samples.

### Isolation of total RNA and cDNA synthesis

Approximately equal sized hemispheres from each rat brain were used for gene microarray analysis (see above for extraction and dissection method). RNA was isolated from frozen brain tissue using TRIZOL (Life Technologies, Carlsbad, CA) and stored at −80 °C. Total RNA quality was assessed using an Agilent 2100 Bioanalyzer (Agilent Technologies, Palo Alto, CA). Sample concentration and purity was assessed using a NanoDrop® ND-1000 Spectrophotometer (NanoDrop Technologies, Wilmington, DE).

From each group, 1 μg of RNA was reverse transcribed into first-strand cDNA using the Invitrogen High Capacity cDNA Reverse Transcriptase Kit (Life Technologies) using random primers. The RT reaction mix (10X RT buffer, 25x dNTP mix, 10X random primers, ThermoFisher Multiscribe reverse transcriptase, RNase inhibitor) was diluted with nuclease free water to a total volume of 20 μl. Reactions were cycled on a GeneAmp PCR System 9700 (Applied Biosystems, Foster City, CA) as follows: 25 °C for 10 minutes, 37 °C for 120 minutes, followed by inactivation at 85 °C for 5 minutes.

### Fluidigm Biomark^TM^ microfluidic qPCR assay

A microfluidic qPCR was performed as previously described^73^. Briefly, for pre-amplification of target genes, 1.25 μl of cDNA template was mixed with 3.75 μl of reaction mix containing 2.5 μl of Preamp Master Mix (Life Technologies) and 1.25 μl of pooled 0.2X TaqMan assays (Applied Biosystems) making for a final pre-amplification reaction volume of 5 μl. Validated TaqMan assay primer sets (Table 2) for each rat gene were obtained. Pre-amplification of target genes was performed using a ABI thermocycler (Applied Biosystems) using the following conditions: 95 °C for 2 minutes to inactivate RT and to activate Platinum Taq polymerase followed by 20 cycles of 15 seconds at 95 °C and 4 minutes at 60 °C, and finally cooled at 4 °C (Fluidigm, Advanced Development Protocol #41: Single-Cell Gene Expression Using SsoFast EvaGreen SuperMix, BioMark^TM^ HD System). Any unincorporated primers were digested according to the Fluidigm protocol using ExoSAP (Affimetrix, Santa Clara, CA) and the gene targets were diluted and loaded into a 48.48 Dynamic array IFC (Fluidigm, San Francisco, CA) using integrated microfluidic circuitry as per manufacturer’s instructions. The 48.48 Dynamic array IFC was loaded using three to five biological replicates for each group with three experimental triplicates for each sample. A serial dilution of commercial reference samples (Universal Rat Reference RNA, Agilent, Santa Clara, CA) was also included directly on the chip to validate primer set amplification conditions. Two sets of separate primers were used for specific genes under investigation. The 48.48 Dynamic array IFC was run using a Biomark^TM^ HD (Fluidigm) according to manufacturer’s instructions and the cycle threshold of each gene was determined by the Cedars-Sinai genomics core.

### Statistical and enrichment analysis

Heavy metal amounts in rat brains after PM exposures were compared to metal contents after exposures to filtered air. A one-way ANOVA and post-hoc t-tests were used to analyze metal accumulation in brains from each exposure condition vs. brains from matched filtered air controls. Statistical significance was set at p < 0.05. Data are expressed as means and their standard error means. Sample sizes were generally 5–6 brains per group.

For qPCR data, the ΔCT method was used to determine fold change between samples. Briefly, the ΔCT for each target gene was determined by subtracting the threshold (CT) value for actin + GAPDH from the CT value for the target gene^74^. The raw CT values for actin and GAPDH were highly comparable. The Welch’s t-test and False Discovery Rate (FDR) test were performed to compare the differences in ΔCT between filtered air and other particulate groups. Data were expressed as mean ± SEM and differences were considered statistically significant if p < 0.05 for the Welch’s t-test and if p < 0.1 for FDR test.

To identify the potential correlations between gene expression (ΔCT) and heavy metal content (ppm), a non-parametric Spearman Rho correlation was performed and the correlation plot was generated by Corrplot Package version 0.77 in R v3.3.1. This analysis assesses how well the relationship between two variables can be described using a monotonic function. Rho values ≥0.5 and ≤−0.5 with p-values of ≤0.05 were considered significant^75^.

### Data Availability Statement

The authors declare that they will make materials, data and associated protocols promptly available to readers without undue qualifications in material transfer agreements.

## Electronic supplementary material

Supplementary Information
