# Supplementary material for: Coarse particulate matter (PM2.5–10) in Los Angeles Basin air induces expression of inflammation and cancer biomarkers in rat brains
Source: Sci Rep. 2018 Apr 9;8:5708. doi: 10.1038/s41598-018-23885-3 (PMC5890281; doi:10.1038/s41598-018-23885-3)
Supplement: Supplementary file 1 — Supplementary Information [file 41598_2018_23885_MOESM1_ESM.pdf]

Supplementary Material

Coarse particulate matter ($PM_{2.5-10}$) in Los Angeles Basin air induces expression of inflammation and cancer biomarkers in rat brains.

Julia Y. Ljubimova[1], Oliver Braubach[1*], Rameshwar Patil[1], Antonella Chiechi[1], Jie Tang[2], Anna Galstyan[1], Ekaterina S. Shatalova[1], Michael T. Kleinman[3], Keith L. Black[1], Eggehard Holler[1,4]

[1] Department of Neurosurgery, Cedars-Sinai Medical Center, Los Angeles, 90048, USA.

[2] Genomics Core, Cedars-Sinai Medical Center, Los Angeles, 90048, USA.

[3] Department of Community and Environmental Medicine Air Pollution Health Effects Laboratory, University of California, Irvine, 92697, USA.

[4] Institut für Biophysik und Physikalische Biochemie der Universität Regensburg, Regensburg, 93040 Germany.

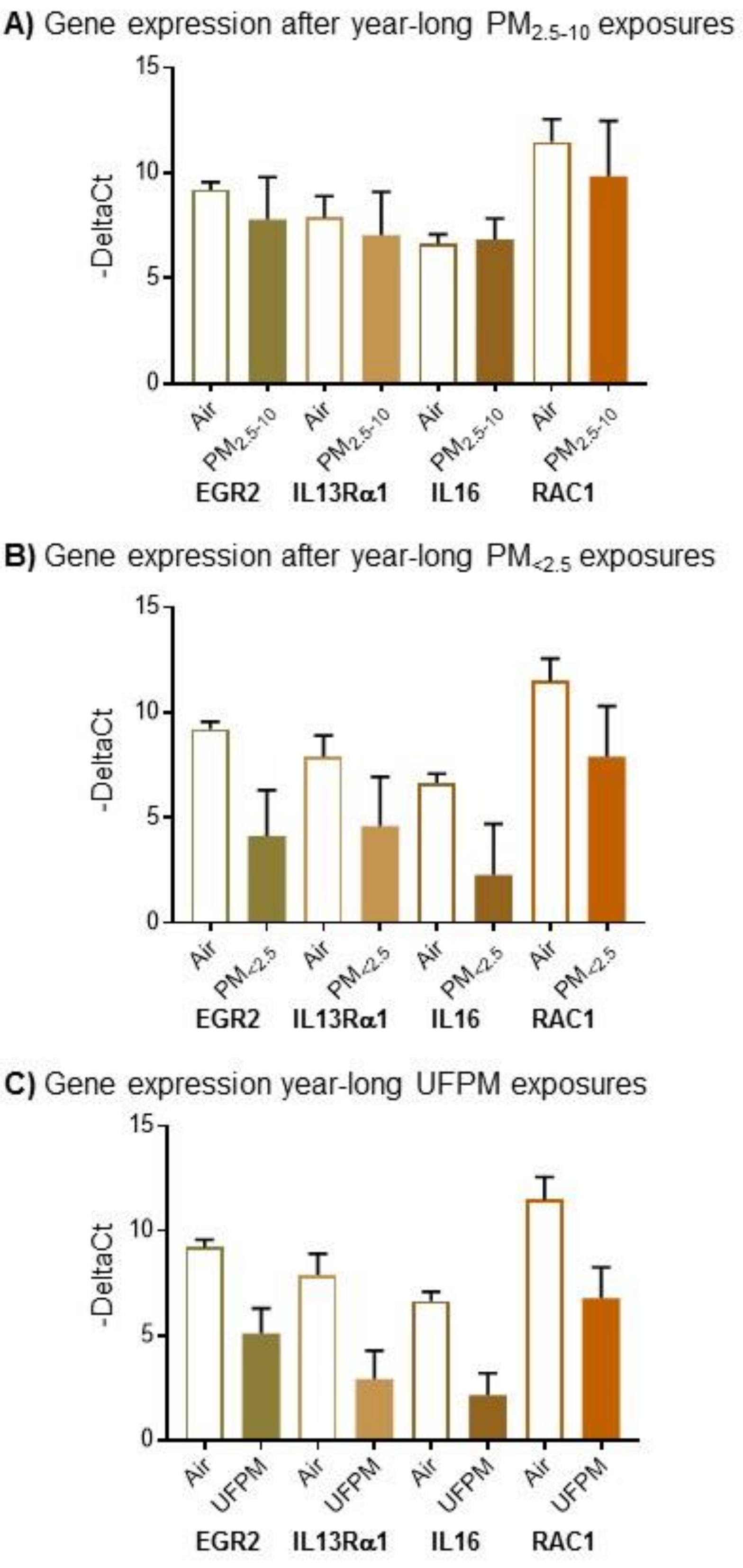

Supplementary Figure S1. Expression of genes related to inflammation (EGR2, IL13Rα1, IL16) and cancer (RAC1) in the rat brain after prolonged exposures to air pollution (1 year). No genes are upregulated in the PM vs. filtered air groups. Data are shown as mean + S.E.M.

Supplementary Table S2. Mass and composition of PM used in this experiment

PM Type	UFPM	$PM_{<2.5}$	$PM_{2.5-10}$
Median Diameter (nm)	70	700	3000
Particle concentration (μg/m³)	63 ± 8	149 ± 24	58 ± 7
Particle number (particles/cm³ 10^{-3})	65 ± 5	67 ± 6	-
EC mass (μg/m³) (%)	5.0 ± 1.1<br>8.0	5.8 ± 1.1<br>3.9	1.7 ± 0.6<br>2.9
OC mass (μg/m³) (%)	17.3 ± 2.8<br>27.5	19.2 ± 2.7<br>12.8	11.8 ± 3.5<br>20.3

PM mass and concentration used for exposure experiments. Experiments were conducted at the same time as those in [26] and the table was adapted from the aforementioned study. The abbreviations EC indicate elemental carbon and OC organic carbon.
